# Lactulose to the Rescue: A Case of Toxic Hepatic Encephalopathy Caused by Portosystemic Shunting and Epistaxis in a Patient with Hereditary Hemorrhagic Telangiectasia

**DOI:** 10.1155/2019/7573408

**Published:** 2019-03-26

**Authors:** Ruchit N. Shah, Michael Makar, Nasir Akhtar, Erin Forster

**Affiliations:** ^1^Geisinger Medical Center, Department of Internal Medicine, 100 N Academy Ave, Danville, PA 17822, USA; ^2^Geisinger Commonwealth School of Medicine, 525 Pine Street, Scranton, PA 18510, USA; ^3^Geisinger Medical Center, Department of Gastroenterology, 100 N Academy Ave, Danville, PA 17822, USA; ^4^Medical University of South Carolina, 30 Courtenay Dr, Charleston, SC 29425, USA

## Abstract

Hereditary hemorrhagic telangiectasia (HHT) is an uncommon autosomal dominant disorder characterized by telangiectasias and arteriovenous malformations. Multiple organ systems are involved including the skin, lungs, gastrointestinal tract, and brain. Hepatic encephalopathy is an extremely rare complication of HHT and early diagnosis and treatment can be life-saving. We present a rare case of hepatic encephalopathy caused by HHT-induced portosystemic shunting treated with lactulose.

## 1. Introduction

Hereditary hemorrhagic telangiectasia (HHT) or Osler-Weber-Rendu syndrome is a rare autosomal dominant vascular disorder characterized by telangiectasias and arteriovenous malformations (AVMs). Multiple organ systems are involved including the skin, lungs, gastrointestinal tract and brain [1]. Common complications of HHT include bleeding disorders such as epistaxis, gastrointestinal bleeding, iron deficiency anemia, and neurologic sequelae including stroke and hemorrhage. Patients with liver involvement commonly present with symptoms of high-output cardiac failure, portal hypertension, and biliary disease [2]. Portosystemic shunting leading to encephalopathy is a rare complication likely secondary to portal hypertension and severe hepatocellular disease [3, 4]. This case highlights the importance of a multisystem approach to patients with HHT and reviews the treatment and management of hepatic complications.

## 2. Case Presentation

**Figure 1:**
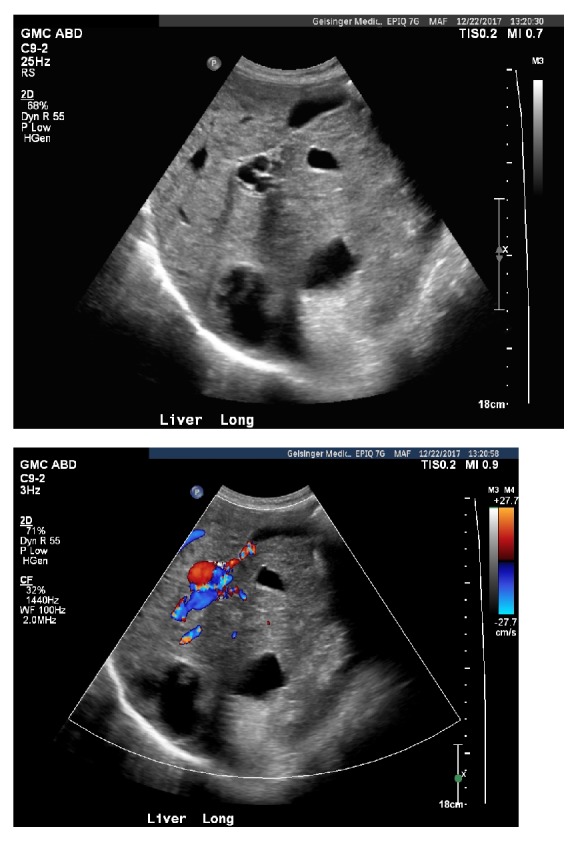
(a) Ultrasound gray scale image shows multiple anechoic structures in the liver with surrounding echogenicity. There is flow on color Doppler (b), consistent with hepatic arteriovenous malformations.

**Figure 2:**
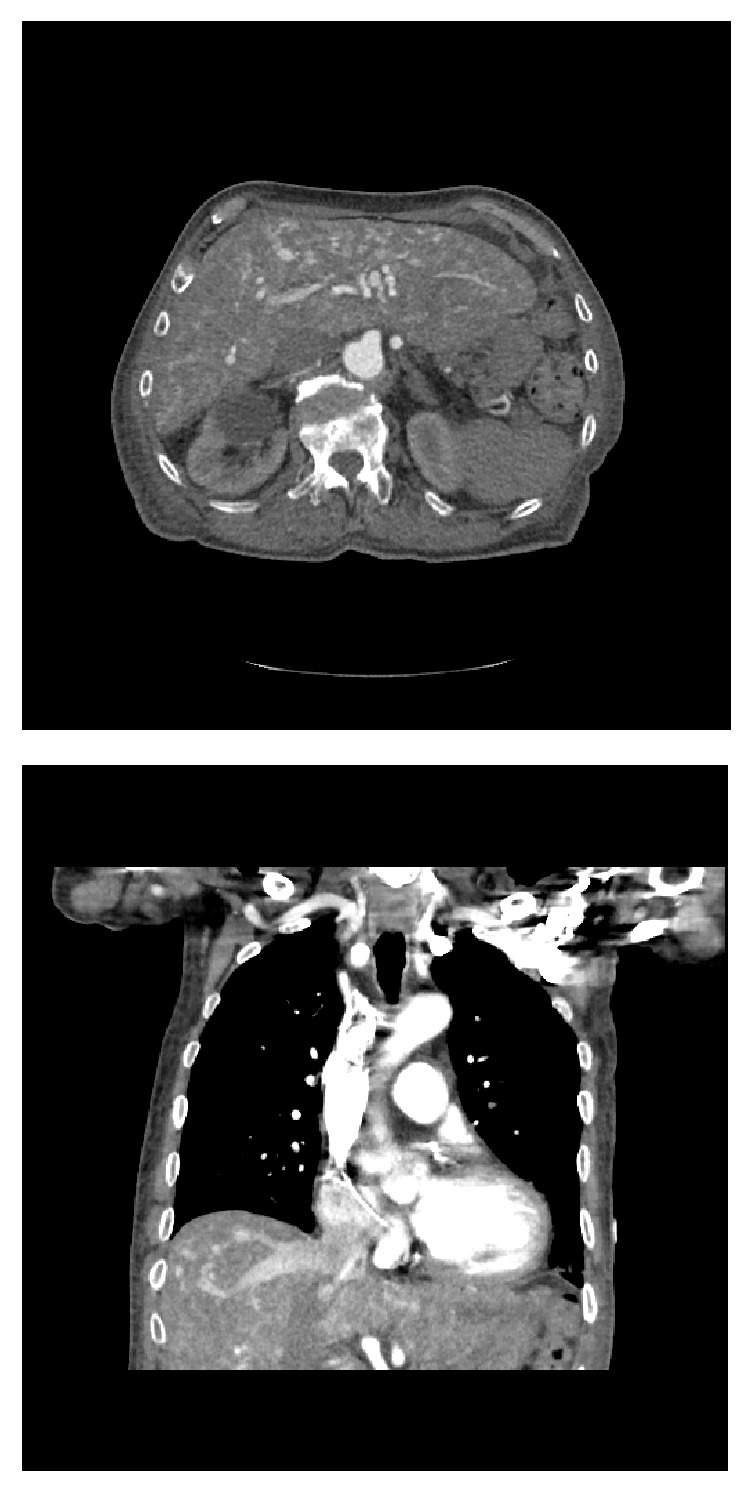
Computed Tomography in the axial (a) and coronal (b) arterial phase images showing an increased number of opacified hepatic arteries with early opacification of draining veins, consistent with multiple hepatic AVMs.

An 85-year-old male with a medical history pertinent for HHT and congestive heart failure presented with epistaxis, altered mental status, and melena. Physical exam revealed a lethargic male with generalized abdominal tenderness, asterixis, and telangiectasias on the lower lip and dorsal aspect of the hands. Ammonia level was 68 umol/L (ref: 11-35 umol/L), alkaline phosphatase 244 U/L (ref: 0-153 U/L), AST 25 and ALT 29 U/L (ref: 10-50 U/L), bilirubin 0.7mg/dL (ref: 0-1.2 mg/dL), INR 4.11 (ref: 0.87-1.17), and hemoglobin 6.8 g/dL (ref: 13.7-16.5 g/dL). Doppler abdominal ultrasound showed coarse liver parenchyma with multiple scattered hyperechoic lesions suggestive of hepatic AVMs (Figure 1). Computed tomography (CT) confirmed the presence of hepatic venous enhancements, consistent with AVMs (Figure 2). The patient was diagnosed with HHT-induced portosystemic encephalopathy secondary to AVMs and recurrent epistaxis. He was started on lactulose with complete resolution of his symptoms. Epistaxis was managed with nasal packing. He remained stable and was discharged.

## 3. Discussion

Hereditary hemorrhagic telangiectasia is a rare inherited autosomal dominant disease that may present with a variety of clinical manifestations. Multiple organ systems are involved including the skin, lungs, gastrointestinal tract and brain [1]. HHT-induced portosystemic encephalopathy (PSE) secondary to AVMs is a rare life-threatening complication.

Clinicians must maintain a high index of suspicion for hepatic encephalopathy in patients with HHT presenting with altered mental status and epistaxis. Symptoms from liver involvement are often misdiagnosed and early identification and treatment in these patients is life-saving. The Curacao criteria are used to diagnose HHT which include three of the following features: recurrent and spontaneous epistaxis, mucocutaneous telangiectasias, visceral involvement, and family history or first-degree relative with HHT [5]. Portosystemic encephalopathy is an extremely rare manifestation of HHT. Buscarini and colleagues studied the clinical manifestations in HHT patients with liver AVMs and reported only two out of one-hundred and fifty-four patients had developed portosystemic encephalopathy [6]. There are three types of portosystemic encephalopathy differentiated based on cause: Type A is associated with acute liver failure, Type B is a consequence of portosystemic shunts with no existing liver disease, and Type C is found in patients with cirrhosis and chronic liver disease [7]. Portosystemic encephalopathy secondary to HHT must be differentiated from other possible causes of encephalopathy related to concomitant conditions such as liver disease due to hepatitis or cirrhosis that may confound the diagnosis. Diagnosis with positive antibody titers for hepatitis B and C and using CT imaging to identify liver disease can be helpful in the initial workup. Further, clinicians must take into consideration the complete clinical picture and precipitating factors such as infections, gastrointestinal bleeding, and electrolyte disorders must be adequately identified and treated. The most common clinical symptoms from HHT are due to bleeding such as epistaxis or gastrointestinal hemorrhage from telangiectasias. Our patient presented with epistaxis and altered mental status and was diagnosed with portosystemic encephalopathy secondary to hepatic AVMs from HHT.

The majority of patients with liver AVMs are asymptomatic, as symptoms secondary to liver AVMs occur in only 5-8% of patients with liver AVMs [8, 9]. Symptomatic patients with hepatic involvement usually present with high-output cardiac failure, biliary ischemia, and portal hypertension [10]. Hepatic vascular malformations are widespread and various patterns of abnormal vascular communications can occur [11]. The three types of vascular shunts in patients with HHT are arteriosystemic shunts between a hepatic artery and a hepatic vein, arterioportal shunts between a hepatic artery and the portal venous system, and portosystemic venous shunts that form between a portal vein and a hepatic vein [12]. Shunts are common complications of hepatic AVMs and may lead to encephalopathy. “Pseudocirrhosis” is described as a morphologic sign in patients with hepatic manifestations of HHT because of the changes that mimic liver cirrhosis following chronic liver diseases. Patients may be misdiagnosed with cirrhosis because of the combination of regenerative nodules and fibrosis but the preservation of normal hepatocellular architecture has led to the term “pseudocirrhosis” [13, 14]. Many clinicians are involved in the care of these patients and being familiar with the various clinical manifestations is imperative for patient care.

Angiography is the gold standard for diagnosing AVMs but less invasive modalities such as Doppler ultrasound, magnetic resonance imaging, and CT imaging may also be used. Diagnosis on CT can be identified through diffuse liver telangiectasias and dilated hepatic arteries [13]. The three types of shunts in HHT are not easily distinguished and conventional imaging cannot easily identify portosystemic shunts. Recently, three-dimensional sonography has been used as a noninvasive method for examining the hepatic vasculature and visualizing portovenous shunts [15]. There are no standard medical therapies for patients with HHT and treatment is patient specific and varies with clinical manifestations. The mainstay of treatment for encephalopathy is medical management with osmotic laxatives. Prior studies have shown successful treatment with lactulose in patients with hepatic encephalopathy [6]. Candelli and colleagues studied the influence of hepatic AVMs on the liver first-pass effect on drugs in HHT patients and found a statistically significant reduced metabolism rate in HHT when compared to controls [16]. Shunt reduction via surgical ligation or transarterial embolization has also been performed in these patients [17]. In patients who are unresponsive to medications, liver transplantation is the only definitive treatment [18]. Systemic treatment options such as Bevacizumab have shown improvement in patients with hepatic complications [19, 20]. This case is an unusual presentation of hepatic encephalopathy caused by HHT-induced portosystemic shunting treated successfully with lactulose.

## 4. Conclusion

In patients with hereditary hemorrhagic telangiectasia and liver involvement, hepatic encephalopathy is a rare and life-threatening presentation. This case highlights the importance of a multisystem approach to patients with HHT and considers the management and treatment of hepatic complications. While HHT and its complications are underdiagnosed, there is an increasing understanding of this disease and its various presentations. This case report presents a patient with hepatic encephalopathy caused by portosystemic shunting in a patient with hereditary hemorrhagic telangiectasia.
